# Transcatheter Closure of Atrial Septal Defect with Carag Bioresorbable Septal Occluder™: First-in-Child Experience with 12-MonthFollow-Up

**DOI:** 10.1155/2022/3476398

**Published:** 2022-12-30

**Authors:** Alessia Callegari, Daniel Quandt, Johannes Nordmeyer, Stephan Schubert, Peter Kramer, Walter Knirsch, Oliver Kretschmar

**Affiliations:** ^1^Division of Pediatric Cardiology, Pediatric Heart Center, and Children's Research Center, University Children's Hospital Zurich, Zurich, Switzerland; ^2^University of Zürich (UZH), Zurich, Switzerland; ^3^Department for Congenital Heart Disease/Pediatric Cardiology, German Heart Institute, Berlin, Germany; ^4^Department of Congenital Heart Disease/Pediatric Cardiology, Heart and Diabetes Center NRW, Ruhr‐University Bochum, Bad Oeynhausen, Germany

## Abstract

**Background:**

Nowadays, transcatheter device closure of an atrial septal defect (ASD) is a standard approach in children. Potential early and long-term side effects or complications related to the metal framework of the devices are a known issue. A bioresorbable device such as the Carag Bioresorbable Septal Occluder™ (CBSO) could resolve such complications.

**Material and Results:**

The Carag Bioresorbable Septal Occluder™ (CBSO; Carag AG, Baar, Switzerland) is a self-centering double disk, repositionable, and retractable device with a bioresorbable framework (polylactic-co-glycolic acid), which is almost completely resorbed by 18–24 months postimplantation. This manuscript reports the four first-in-child ASD device closures using a CBSO. The patients' age was median (IQ1-IQ3), 4.5 years (4–7.25). Weight was 21.3 kg (17.6–32.7). We demonstrated procedural feasibility and safety. Effective defect closure with the device was 100%. Echocardiographic measurements of the thickness of the interatrial septum did not show any relevant increase over a 12-monthfollow-up period. There were no residual defects found after the procedure or later during the resorption process. The patients showed no evidence of any local or systemic inflammatory reaction.

**Conclusions:**

The CBSO device system could offer a new treatment option for transcatheter ASD device closure in the pediatric and adult fields. In our first-in-child experience, it was effectively and safely implanted. During the first 12 months of follow-up, no complications occurred.

## 1. Introduction

Routine transcatheter device closure of secundum type atrial septal defect (ASD II) has been performed for more than twenty-five years, with excellent long-term results [1]. The success rate using different types of ASD closure devices is more than 98%, but devices with metal frameworks can rarely cause serious complications such as erosion, perforation, thrombus formation, inflammation, blood flow disturbance, conduction disturbance, and atrial arrhythmia during long-term follow up due to the metal properties of the implant [1–3]. An atrial septal occluder with a bioresorbable framework could potentially reduce the incidence of such complications, and the Carag Bioresorbable Septal Occluder™ (CBSO) is the first interatrial septal occluder that fulfills these characteristics [4, 5]. While the safety and effectiveness of the CBSO device have been reported in preclinical animal studies and in adult studies [2, 4–6], this is the first to report of children treated with this new device.

## 2. Methods

### 2.1. Patients and Device Description

We report on the 4 first-in-child patients with transcatheter ASD closure using the CBSO. These 4 patients were treated at the University Children's Hospital of Zurich, Switzerland, and at the German Heart Institute in Berlin, Germany. At the time of implantation, the CBSO device had European CE Mark approval, and in addition to the regular consent for the interventional procedure, all guardians gave their informed consent for inclusion in the “Multicenter, international, postmarket registry to monitor the clinical performance and safety of an atrial septal closure device with a bioresorbable framework in patients with a clinically significant atrial septum defect (ASD) or patent foramen ovale (PFO)” (Nr°: 2019-01836). The study was conducted in accordance with the Declaration of Helsinki.

**Figure 1:**
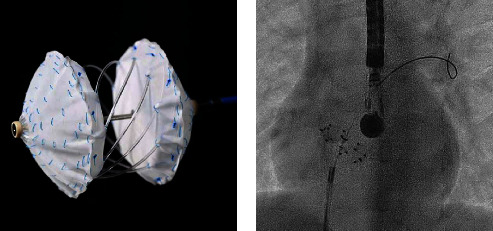
Schematic representation of the CBSO occluder (a). The device has two opposing polyester covers that are attached to a PLGA monofilament framework. At each end of the filaments (a total of 8), there is a filament holder made of polyetheretherketone. At the distal tip of the implant, a nut made of Phynox keeps the filaments in place and ensures visibility under X-ray (b).

The Carag Bioresorbable Septal Occluder™ (CBSO; Carag AG, Baar, Switzerland) is the first occluder where the metal framework is replaced with bioresorbable polylactic-co-glycolic acid (PLGA). It is to note that the CBSO is now the “reSept™ ASD Occluder,” being developed by atHeart Medical™ AG, Baar, Switzerland. It is a self-centering device with two opposing polyester covers attached to a PLGA monofilament framework. At each end of the filaments (a total of 8 or 10, depending on the size of the occluder), there is a filament holder made of polyetheretherketone (PEEK), a nonresorbable polymer. At the distal tip of the implant, a nut made of Phynox (a cobalt-chromium-nickel alloy) keeps the filaments in place and ensures, together with platinum-iridium markers, excellent visibility under X-ray (Figure 1). Bioresorption begins after 6 months, and the device is almost completely resorbed by 18–24 months postimplantation [2, 4]. Its endothelialization should be completed within 3 months [2, 4].

The occluder is delivered through a 12 Fr long transseptal sheath over a 0.018″ guidewire. The delivery system consists of two coaxial control catheters that permit independent control of the proximal and distal ends of the device, allowing for traditional deployment, with the right atrial disk delivered first, or both the left and right atrial disks in parallel. This implantation technique has been proven feasible and was described in detail for the Solysafe septal occluder in pediatric patients before [7, 8]. The occluder is fully retrievable and redeployable at all stages of delivery [4, 5].

For the postmarket registry, there were 3 device sizes, which allowed for defect closure up to 25 mm. Two sizes of CBSO were used: type S with an outer disc diameter of 26 mm (to treat defects from 4 to 12 mm) and type M with an outer disc diameter of 28 mm (to treat defects from 11 to 20 mm).

We included patients with rims of at least 4-5 mm, and these were carefully evaluated with echocardiography prior to and during the intervention.

### 2.2. Periprocedural and Postprocedural Treatment

All procedures were performed according to our institutional standards. Patients were under general anesthesia, and closure was performed under transesophageal echocardiography (TOE) and fluoroscopic guidance in accordance with the instructions for use. Balloon sizing of the defect has been performed in three patients, while in the remaining patient, we exclusively measured the dimensions of the defect at transesophageal echocardiography. For anticoagulation management, standard heparin (100 IU/kg) was administered intravenously, followed by subcutaneous application of low molecular weight heparin (1 mg/kg) for 36 hours postprocedure. After the defect was closed, all patients received aspirin 3–5 mg/kg/day for six months. Periprocedural antibiotic prophylaxis (cefazolin, 25 mg/kg) was given at the start of the procedure and continued for 3 doses.

## 3. Results

### 3.1. Patients and Procedures

The patients' ages were median (IQ1-IQ3) 4.5 years (4–7.25), with 2 females and 2 males. Weight was 21.3 kg (17.6–32.7). Preinterventionally, all patients had dilated right-sided heart structures on an echocardiogram without any clinical distress. All patients had one central defect (oval in two patients and round in two patients), and the defect diameter measured 11 mm (9.2–12.7). Periprocedural (TOE) septal thickness (close to the defect) on the short-axis view was 3.6 mm (3.4–3.7) and 3.9 mm (3.5–4.2) on the 4-chamber views. The closure was performed twice with a CBSO-Type-M device and twice with a CBSO-Type-S device. Peri-interventional TOE showed no residual shunt and a correct, flat device configuration in all patients. No peri-interventional or postinterventional complications occurred.

### 3.2. Postprocedural Course and Follow-Up (Figure 2)

At 6 and 12 months of follow-up, all patients had no signs of systemic or local inflammation. The device showed an optimal position and configuration on a transthoracic echocardiogram (TTE) without a residual shunt. The septal thickness on the short-axis view was 4.4 mm (4.1–4.7) and on the 4-chamber view, 4.7 mm (4.2–5.3). The septal thickness increase was 0.9 mm (0.72–1.0) on the short-axis view and 0.65 mm (0.25–1.37) on the 4-chamber view, which reflects the thinness of the device. A 24 h Holter-ECG foundno arrhythmia in all patients.

## 4. Discussion

Usage and handling of this new Carag Bioresorbable Septal Occluder™ (CBSO) were easy and resulted in successful ASD closure in all reported cases. This is in line with the “first-in-man” results of adult patients, in which the success rate for the implantation of the device was 88% [5]. In our four pediatric patients with a medium-sized atrial septal defect, the periprocedural and follow-up (12 months) success rates of defect closure were 100%.

There are two studies that assess the safety of the CBSO device: one is a preclinical assessment [4] and two are pilot clinical studies [5]. The first-in-men clinical study [5] involves 17 adults, and in this cohort, no erosion, perforation, embolization, myocardial infarction, stroke, or interference with the valvar apparatus occurred. One right atrial thrombus was resolved with anticoagulation, and one patient reported palpitations. In the preclinical study [4], all devices were macroscopically intact, and no device-related complications occurred within the observation time (15 months). Similarly, none of our patients experienced complications during the peri-interventional or during the 12-monthfollow-up.

An atrial septal occluder with a bioresorbable framework is very attractive for usage in pediatric patients since it leaves an endothelialized “natural” atrial septum with intact growth potential behind [9]. Furthermore, we are confident that this device could reduce the burden of mid- and long-term complications arising from the metal frameworks of other current devices. Degradation of the PLGA filaments with substitution of the polymer material by fibromuscular cells and extracellular matrix in a similar pattern compared to neighboring tissue parts was demonstrated to proceed with time [4].

Therefore, it is reasonable to assume that this could permit the later interventional crossing of the atrial septum to access the left side of the heart for future interventions [9]. Nevertheless, the clinical feasibility of a transseptal puncture has not been demonstrated yet.

This self-centering device showed a flat configuration without obstruction or erosion of the neighboring cardiac structures directly after implantation and at a 12-monthfollow-up in all our patients.

Usage of the CBSO types S and M allowed successful closure of atrial septal defects with a diameter ranging from 6 to 16 mm, suggesting possible implantation in (at least) small to medium-sized defects in children [5]. Due to the moderately stiff and straight device delivery system, a lacking retro-aortic rim (<4-5 mm) could potentially be an important issue, resulting in failure of device placement. In fact, the discs of the device are configured with the center of the device in the defect, and it could be difficult to deploy the left atrial disc properly behind the aorta and to prevent prolapse into the right atrium. The option to deploy the right atrial disc and push this configured disc towards the septum before the left atrial side might help to overcome this issue. Nevertheless, this maneuver was not attempted in our first four patients.

There were no clinical signs of systemic or local inflammation due to the ingrowth, reabsorption, and endothelialization processes of the device in our population [2, 4]. The thickness of the interatrial septum, as a marker of local tissue reaction and inflammation, measured on serial echocardiograms, did not increase significantly from implantation to the 12-monthfollow-up.

Under routine antiplatelet therapy with aspirin during the first 6 months after implantation, no thrombus formation could be detected.

## 5. Conclusions

From our first experience in children, we can assume that transcatheter implantation of the CBSO is safe with complete closure of the defects during the procedure and at the 12-monthfollow-up. Lacking retro-aortic rim (<4-5 mm) could potentially remain anissue resulting in failure of device placement. Additional patients and longer follow-up are needed to further assess the outcome and the impact on long-term complications, as well as the feasibility of left-sidedtrans-atrial catheter interventions via transseptal puncture after complete device reabsorption.

## Figures and Tables

**Figure 2:**
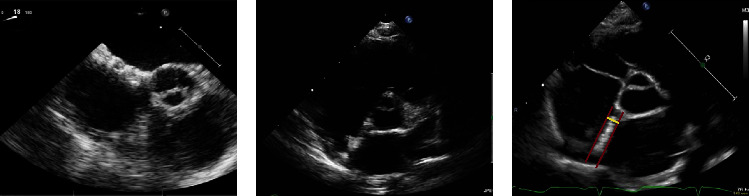
Short-axis view showing the visibility of the implanted CBSO occluder during implantation (on TOE), at 6 months follow-up (on TTE), and at 12 months follow-up (on TTE). The site of measurement of a septal thickness (yellow line) is shown in the image on the right. (a) Periprocedural (TOE), (b) at 6 months follow-up (TTE), and (c) at 12 months follow-up (TTE).

## Data Availability

The data used to support the findings of this study are available within the article. Supplementary data that support the findings of this study are available from the corresponding author upon reasonable request.
